# Moderate and severe TBI in children and adolescents: The effects of age, sex, and injury severity on patient outcome 6 months after injury

**DOI:** 10.3389/fneur.2022.741717

**Published:** 2022-08-03

**Authors:** Lori Kennedy, Miriam Nuno, Gene G. Gurkoff, Kristin Nosova, Marike Zwienenberg

**Affiliations:** ^1^Center for Nursing Science, University of California Davis Health, Sacramento, CA, United States; ^2^Public Health Sciences, Medical Sciences 1-C, University of California, Davis, Davis, CA, United States; ^3^Department of Neurological Surgery, University of California, Davis, Davis, CA, United States; ^4^Center for Neuroscience, University of California, Davis, Davis, CA, United States

**Keywords:** outcome, prediction model, traumatic brain injury, pediatric, GCS-P, abusive head trauma, secondary injury

## Abstract

The interaction of age, sex, and outcomes of children with head injury remains incompletely understood and these factors need rigorous evaluation in prognostic models for pediatric head injury. We leveraged our large institutional pediatric TBI population to evaluate age and sex along with a series of predictive factors used in the acute care of injury to describe the response and outcome of children and adolescents with moderate to severe injury. We hypothesized that younger age at injury and male sex would be associated with adverse outcomes and that a novel GCS-based scale incorporating pupillary response (GCS-P) would have superior performance in predicting 6-month outcome. GCS and GCS-P along with established CT scan variables associated with neurologic outcomes were retrospectively reviewed in children (age birth to 18 years) with moderate or severe head injury. GOS-E was prospectively collected 6 months after injury; 570 patients were enrolled in the study, 520 with TBI and 50 with abusive head trauma, each analyzed separately. In the TBI cohort, the median age of patients was 8 years and 42.7% had a severe head injury. Multiple predictors of outcome were identified in univariate analysis; however, based on a multivariate analysis, the GCS was identified as most reliable, outperforming GCS-P, pupil score, and other clinical and CT scan predictors. After stratifying patients for severity of injury by GCS, no age- or sex-related effects were observed in our patient population, except for a trend toward worse outcomes in the neonatal group. Patients with abusive head trauma were more likely to have severe injury on presentation, increased mortality rate, and unfavorable outcome. Additionally, there was clear evidence that secondary injuries, including hypoxia, hypotension, and hypothermia were significantly associated with lower GCS and higher mortality in both AHT and TBI populations. Our findings support the use of GCS to guide clinical decision-making and prognostication in addition to emphasizing the need to stratify head injuries for severity when undertaking outcome studies. Finally, secondary injuries are a clear predictor of poor outcome and how we record and manage these events need to be considered moving forward.

## Introduction

Traumatic brain injury (TBI) is a leading cause of death and disability, contributing to one-third of all injury-related deaths in the U.S. (1). Despite extensive research efforts in randomized controlled trials (RCTs), advances in prognosticating outcome after injury have been limited. Although several studies demonstrated that age and sex influence outcome, these factors have not been rigorously evaluated in the development of prognostic indicators. Analysis of existing trauma databases may elucidate factors associated with worse outcomes after TBI and, in turn, inform early discussion regarding prognosis and anticipated resources needed after injury, as well as influence future clinical trial design.

Prior studies suggest that younger children are more likely to have worse long-term outcomes after TBI (2–7). This is thought to be due to the increased vulnerability of the developing brain and the subsequent developmental lag that occurs, especially in very young children (8). Moreover, there is evidence that there are unique characteristics of the pediatric skull and brain that change over the course of development, altering the biomechanics of injury and potentially affecting outcome independent of brain development (9, 10).

The roles of both sex and gender in TBI are less well-understood. Sex refers to the biological and physical characteristics of the male and female bodies and includes anatomical, genetic, physiological, and hormonal characteristics (11). Gender is a socio-cultural-based construct referring to what is socially labeled “feminine” or “masculine” and how these qualities are expressed. Both sex and gender can influence the clinical outcome of TBI and influence specific domains such as social integration and cognitive performance (12, 13). Multiple factors contribute to the observed differential in outcomes including neuroprotective effects of female sex hormones and a different microglial inflammatory response in male vs. female brains, as well as various strategies females may use to cope with the social impairment that occurs after TBI (1, 14–17).

We leveraged our large pediatric TBI population to evaluate the association of novel and established predictive factors routinely collected in the acute care phase of injury with 6-month outcomes among children with moderate-to-severe TBI. We assessed the impact of age and sex on outcomes after injury and evaluated the performance of a novel clinical severity scale adapted from the Glasgow Coma Scale (GCS) and pupillary response, the GCS-P, in a pediatric population. We hypothesized that age at injury and sex would each be significant predictors of outcomes at 6 months and that GCS-P would be a superior predictor of outcome in comparison to the GCS.

## Materials and methods

### Eligibility criteria and study design

This cohort study included 570 patients of age 18 years or younger who experienced a moderate or severe traumatic brain injury and had follow-up outcomes evaluated at 6 months post-injury. Of these, 50 patients experienced abusive head trauma (AHT) and were analyzed separately. At our institution a specialized AHT team evaluates the patients and criteria for making a diagnosis of AHT includes the presence of retinal hemorrhages, (healing) skeletal fractures, and clinical and imaging findings inconsistent with the reported injury. Only patients for whom a definitive diagnosis of AHT was made after evaluation by the AHT team were included in the AHT analysis.

**Figure 1:**
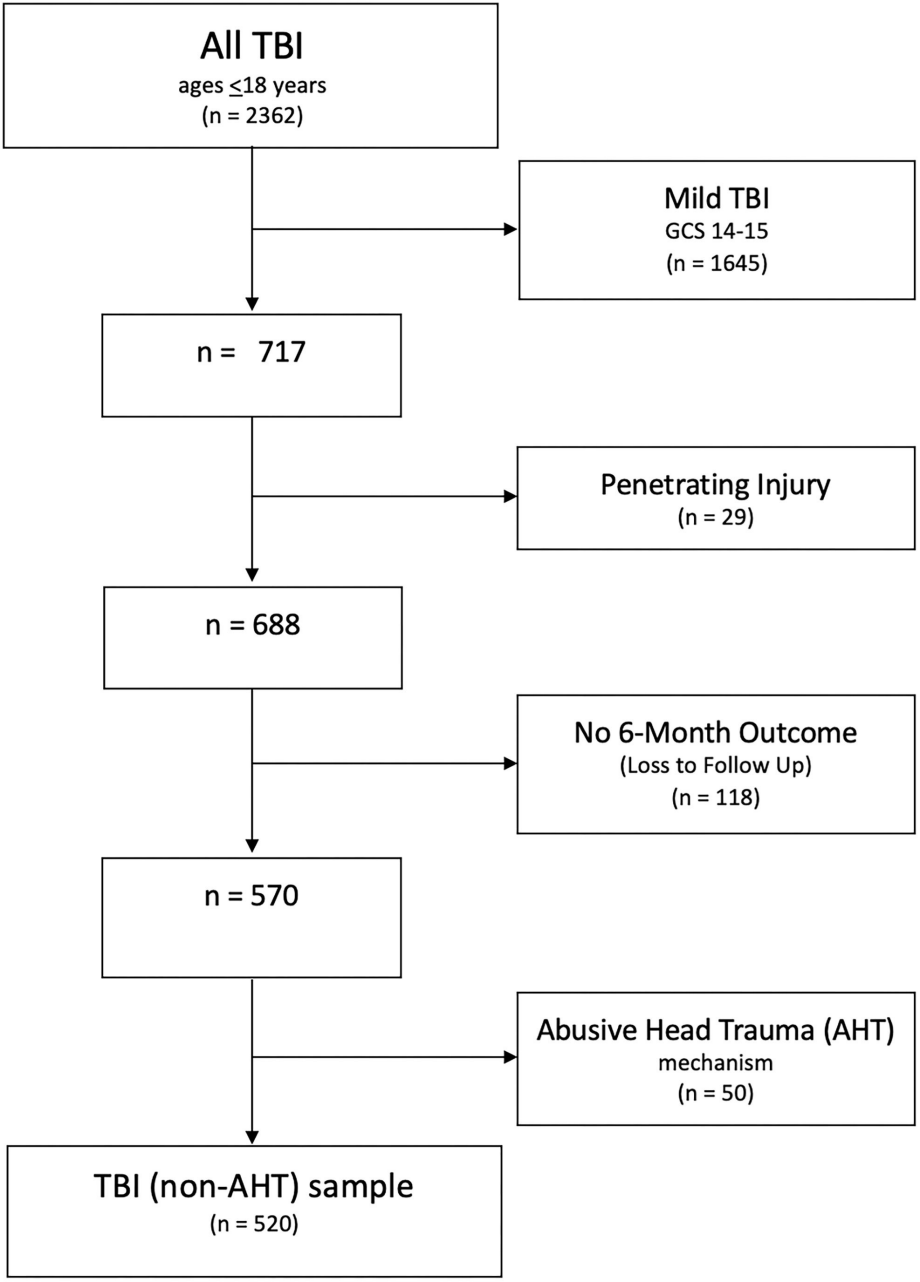
Inclusion decision tree.

All patient data were prospectively collected at our Level 1 Trauma Center and entered into our TBI registry—between 1 January 2008, and 31 December 2020. All pediatric patients with non-penetrating injuries, a positive head CT (any intracranial finding or cranial vault injury), a post-resuscitation GCS score ≤ 13, and a documented GOS-E outcome at 6 months were included in the analysis (Figure 1). Cases with mild (GCS 14-15) and penetrating injuries were excluded to minimize sample heterogeneity, as they represent a very different pattern of injury and overall outcomes (18). Study procedures commenced following IRB approval of the current protocol, IRB number #1663970.

### Data collection

Dedicated unblinded data abstractors entered the admission data into the registry within 24 h of admission. Abstracted variables included age, sex, vital signs, post-resuscitation GCS and its components, pupillary exam, cranial CT findings, mechanism of injury, and secondary injuries such as hypoxia, hypotension, and hypothermia. Cranial CT findings were interpreted by the on-call neurosurgeon and radiologist. Missing or incomplete registry data were supplemented with the information from the electronic medical record; a data audit was conducted on approximately 10% of the records. After inspection for accuracy, the data were deidentified and exported into a Microsoft Excel file for Mac (version 15.23.2, Microsoft Corp.).

### Patient management

All patients were managed following a protocol based on the most current Guidelines for the Management of Pediatric Severe Traumatic Brain Injury (19, 20). Detailed ICU data regarding frequency or type of secondary insults and specific ICU interventions undertaken by providers were not captured in the database.

#### Glasgow coma scale

Post-resuscitation GCS was used to classify the severity of injury with a GCS of 14 to 15 defined as a mild, GCS 9 to 13 as moderate, and a GCS 3 to 8 as severe (21–23). In calculating the GCS of children younger than 2 years of age, the following verbal categories were used: 5. Smiles, oriented to sounds, follows objects, interacts; 4. Cries but consolable, inappropriate interactions; 3. Inconsistently inconsolable, moaning; 2. Inconsolable, agitated; 1. No verbal response. The Motor categories were as follows: 6. Moves spontaneously or purposefully; 5. Withdraws from touch; 4. Withdraws from pain; 3. Abnormal flexion to pain (decorticate response); 2. Extension to pain (decerebrate response); 1. No motor response.

#### GCS-P

GCS-P is a metric that combines the components of GCS with the pupillary exam as measured by a pupil reactivity score (PRS). It was recently developed for the adult population based on the CRASH and IMPACT databases and increased the accuracy of outcome prediction (24). PRS is defined as follows: 0 = both pupils are reactive, 1 = one pupil is reactive, 2 = bilateral pupils are non-reactive. GCS-P is calculated by subtracting PRS from GCS, with the resulting possible score range of 1 to 15. We assessed the performance of GCS vs. GCS-P in the accuracy of outcome prediction and used GCS-P as a categorical variable in our analysis.

#### Age groups

To account for the variability in the response to injury across the pediatric age range, we evaluated age as both a continuous and categorical variable. Following recommendations put forward by Williams et al. (25), age groups were defined as follows: Neonates and infants: 0 to 12 months (inclusive), Toddler: 13 months up to 2 years, Early childhood: 2 to 5 years (inclusive), Middle childhood: 6 to 11 years (inclusive), and Early adolescence: 12–18 years (inclusive) (25).

#### GOS-E

The validated Glasgow Outcome Scale–Extended (GOS-E) at 6 months is the primary outcome measure of this study (26). GOS-E was prospectively collected 6 months after injury *via* structured telephone interviews by specifically trained personnel. The interviewers were blinded to the initial severity of the injury and not involved in the acute care of these patients. Interviews were conducted within 1 week of the 6-month post-injury window.

The GOS-E has eight tiers of recovery [(1) death, (2) vegetative state, (3) lower severe disability, (4) upper severe disability, (5) lower moderate disability, (6) upper moderate disability, (7) lower good recovery, and (8) upper good recovery] and identifies areas such as independence in and outside the home, functioning at school, and ability to maintain social relationships as critical areas to assess recovery after injury (26). While less discriminating than the full scale, it is common practice in studies of TBI to use a dichotomized GOS (27). To compare our findings to other studies, we have used this dichotomized approach for many of our analyses. For this assessment, we categorize patients with moderate disability (lower and upper) and good recovery (lower and upper) as favorable and the remainder of patients as having an unfavorable outcome. For our mortality analysis, we used the GOS-E (1) outcome.

### Statistical analysis

We assessed differences in outcomes at 6 months by patient and injury characteristics using Chi-square and Fisher's exact tests when applicable for categorical variables. For continuous variables, we used Mann–Whitney U tests. We used binary logistic regression to calculate the receiver operator characteristic (ROC) curves (Mann–Whitney U test) that evaluate the performance of GCS and GCS-P for predicting mortality and an unfavorable outcome at 6 months. The models were developed in a nested fashion with a simple model initially including GCS or GCS-P and then increased their complexity with the addition of relevant factors. The ROC provides a measure of diagnostic accuracy, namely the area under the curve (AUC), with a value of 0.5 or lower indicating poor discrimination. The Somers' D statistic, a predictor performance indicator, is provided for each model evaluated. Analyses were conducted for AHT separately from other mechanisms of traumatic brain injury. Covariate-adjusted logistic regression was used to account for observed confounders, and two-way interactions were considered to assess functions affecting the exposure–outcome relationship. All data were analyzed using SAS version 9.4 (SAS Institute, Cary, NC, USA).

## Results

From 2008 to 2020, 688 patients (age 0–18 years) were admitted to the UC Davis Children's Hospital with a blunt moderate or severe head injury. The median age was 8 years (IQR 3-14). At 6 months, 17% of patients were lost to follow-up, leaving 570 patients available for analysis. Patients with AHT (*n* = 50) were analyzed separately from the remaining cohort (*n* = 520*)*.

### Mortality analysis

#### Age and sex

The average mortality rate for patients with TBI was 13.3%, with a slightly higher rate noted in neonates (19.4%) and a lower rate in early childhood (7.2%); however, these differences did not reach statistical significance (*p* = 0.3623). Univariate analysis found no significant difference in mortality rates by sex (Table 1).

**Table 1:** Cohort characteristics by outcomes (GOS-E) at 6 months follow-up (*n* = 520).

**Outcomes at 6 months (GOS-E)**					
**Variables**	**All**	**Died** ***n** =* **77 (13.3)**	***p*** **-value**	**Unfavorable outcome** ***n** =* **178 (31.5)**	***p*** **-value**
Age group^*^			0.3623		0.2533
Neonates and infants	36 (6.9)	7 (19.4)		14 (38.9)	
Toddler	68 (13.1)	9 (13.2)		21 (30.9)	
Early childhood	97 (18.7)	7 (7.2)		20 (20.6)	
Middle childhood	132 (25.4)	14 (10.6)		38 (28.8)	
Early adolescence	187 (36.0)	22 (11.8)		57 (30.5)	
Sex			0.3517		0.7836
Male	342 (65.8)	42 (12.3)		100 (29.2)	
Female	178 (34.2)	17 (9.6)		50 (28.1)	
Mechanism			<0.0001		<0.0001
ATV accident/Fall	182 (35.0)	6 (3.3)		30 (16.5)	
Assault/Kick/ Struck	60 (11.5)	6 (10.0)		13 (21.7)	
Auto vs. Pedestrian	89 (17.1)	19 (21.4)		36 (40.5)	
MCA/MVA	189 (36.4)	28 (14.8)		71 (37.6)	
GCS^**^			<0.0001		<0.0001
moderate	298 (57.3)	1 (0.3)		27 (9.1)	
severe	222 (42.7)	58 (26.1)		123 (55.4)	
Motor response			<0.0001		<0.0001
median (IQR)	5 (4–6)	1 (1–2)		3 (1–5)	
Pupil response			<0.0001		<0.0001
BNR-2	55 (10.6)	40 (72.7)		49 (89.1)	
UNR-1	27 (5.2)	7 (25.9)		20 (74.1)	
BR-0	438 (84.2)	12 (2.7)		81 (18.5)	

* *Age group: Neonates and infants (0–12 months), Toddler (13 months to 2 years), Early childhood (2–5 years), Middle childhood (6–11 years), Early adolescence (12–18 years)*.

** *Severe Head Injury: GCS 3-8, Moderate Head Injury: GCS 9-13*.

#### Clinical severity of injury: GCS/PRS score

The majority of patients who died (75.3%) had severe TBI defined by GCS. However, of the total population with a severe TBI, the mortality rate was 26.1%, whereas patients with moderate TBI had significantly lower mortality (0.3%; *p* < 0.0001). There was also a significant relationship between pupillary reaction and mortality, where mortality was low (2.7%) for patients with bilateral reactive pupils, increasing to 25.9% with one non-reactive pupil and to 72.7% if both pupils were non-reactive (*p* < 0.0001, Table 1).

#### Mechanism of injury

The highest mortality rate (21.4%) was in pedestrians hit by a vehicle, significantly higher than the average mortality rate of the entire sample (*p* < 0.0001, Table 1). The mortality rate in an assault-type injury was similar to a motor vehicle accident/motorcycle accident (MVA/MCA, injured person was in or ejected from a vehicle). Observed mortality after a fall was very low (*n* = 6, 3.3%). Most patients who died from their falls were found to have high-energy injuries, such as a fall from a bridge as opposed to a fall from a crib.

#### Secondary injury

The presence of hypotension, hypoxia, and hypothermia resulted in significant increases in mortality (each *p* < 0.0001). The presence of any of these factors increased the observed mortality to 37.5, 39.4, and 30.8%, respectively *(*Table 2*)*, as compared to 13.3% for the entire population. Of the 31 patients who presented with all three of these secondary injuries, 74.2% died.

**Table 2:** Injury characteristics by outcomes at 6-month follow-up (*n* = 520).

**Outcomes at 6 months (GOS-E)**					
**Variables**	**All**	**Died** ***n** =* **77 (13.3)**	***p*** **-value**	**Unfavorable outcome** ***n** =* **178 (31.5)**	***p*** **-value**
Hypotension			<0.0001		<0.0001
Yes	112 (21.5)	42 (37.5)		65 (58.0)	
No	408 (78.5)	17 (4.2)		85 (20.8)	
Hypoxia			<0.0001		<0.0001
Yes	71 (13.6)	28 (39.4)		48 (67.6)	
No	449 (86.4)	31 (6.9)		102 (22.7)	
Hypothermia^*^			<0.0001		<0.0001
Yes	143 (27.5)	44 (30.8)		67 (46.9)	
No	375 (72.1)	13 (3.5)		81 (21.6)	
Contusion			0.9762		0.9445
Yes	105 (20.2)	12 (11.4)		30 (28.6)	
No	415 (79.8)	47 (11.3)		120 (28.9)	
IVH			<0.0001		<0.0001
Yes	28 (5.4)	10 (35.7)		20 (71.4)	
No	492 (94.6)	49 (10.0)		130 (26.4)	
IPH			0.9872		
Yes	115 (22.1)	13 (11.3)		42 (36.5)	0.0395
No	405 (77.9)	46 (11.4)		108 (26.7)	
SDH			0.0158		0.0009
Yes	232 (44.6)	35 (15.1)		84 (36.2)	
No	288 (55.4)	24 (8.3)		66 (22.9)	
EDH			0.0305		0.2907
Yes	98 (18.9)	5 (5.1)		24 (24.5)	
No	422 (81.2)	54 (12.8)		126 (29.9)	
TSAH			0.0113		0.0316
Yes	195 (37.5)	31 (15.9)		67 (34.4)	
No	325 (62.5)	28 (8.6)		83 (25.5)	
Cisterns compressed/absent			<0.0001		<0.0001
Yes	32 (6.2)	21 (65.6)		29 (90.6)	
No	488 (92.8)	38 (7.8)		121 (24.8)	
Depressed skull			0.2606		0.5990
Yes	47 (9.0)	3 (6.4)		12 (25.5)	
No	473 (91.0)	56 (11.8)		138 (29.2)	
MLS			0.0078		<0.0001
Yes	466 (89.6)	12 (22.2)		31 (57.4)	
No	54 (10.4)	47 (10.1)		119 (25.5)	

* *2 missing*.

#### CT imaging characteristics

Epidural hematoma on head CT was associated with significantly fewer deaths (5.1%, *p* = 0.0305) than cases in which SDH was identified (15.1%, *p* < 0.05). Neither the presence of intraparenchymal hemorrhage (*p* = 0.9872) nor depressed skull fracture (*p* = 0.2606) was associated with increased mortality. The presence of traumatic SAH, IVH (*p* < 0.0001), compressed or absent cisterns (*p* < 0.0001), and midline shift (*p* = 0.0078) were associated with increased mortality; two-thirds of patients with compressed or absent cisterns died.

### Six-month outcomes analysis

All data were first analyzed using the full GOS-E scale. However, as the GOS-E subgroups were too small, with at most 65 patients in one subgroup and only 5 in another, we could not generate meaningful comparisons. Therefore, we collapsed the GOS-E data into two categories, favorable (UGR/LGR/UMD/LMD) and unfavorable outcome (USD/LSD/V/D) for analysis.

#### Age and sex

Based on GOS-E, children in the early childhood group had the lowest rates of unfavorable and the highest rate of a favorable outcomes, although the differences were not statistically significant. Similarly, we did not find any sex-related difference in the rates of unfavorable outcomes (*p* = 0.7836) or allocation to best or worst prognosis groups (Table 1).

#### Mechanism of injury

Patients with Auto vs. Pedestrian injuries had the highest rate of unfavorable outcomes (40.5%), followed by patients injured in an MVA or MCA (37.6%). In patients with falls and assault-type injuries, the rate of unfavorable outcomes was much lower (16.5 and 21.7%, respectively) (Table 1).

#### Secondary injury (hypoxia, hypotension, and hypothermia)

The secondary injury was identified in admission data. Hypoxia is coded for two consecutively documented SpO_2_ values <90% and/or an arterial blood gas PaO_2_ <60%. Hypotension is coded if the systolic blood pressure is <90 mmHg [or for patients aged 9 years or younger, <70 mmHg + 2x (“x” is the age of the patient)]. The first temperature documented in the electronic health record is recorded with values <36°C coded as hypothermia.

Secondary injury increased the rate of unfavorable outcomes significantly: in patients with hypotension (*p* < 0.0001) or hypoxia (*p* < 0.0001), a 3-fold increase in unfavorable outcomes was noted. Patients with hypothermia were twice more likely to experience an unfavorable outcome (*p* < 0.0001, Table 2). Of the 31 patients who presented with all three of these secondary injuries, 87.1% had unfavorable outcomes.

#### Clinical severity of injury: GCS, GCS-P = GCS—pupil score

Fewer than 10% of patients with moderate head injury (GCS 9-13) had an unfavorable outcome (*p* < 0.0001). In contrast, patients with a severe head injury (GCS ≤ 8) were six times more likely to have an unfavorable outcome (55%, *p* < 0.0001) and 48% had a lower recovery (Table 1).

**Figure 2:**
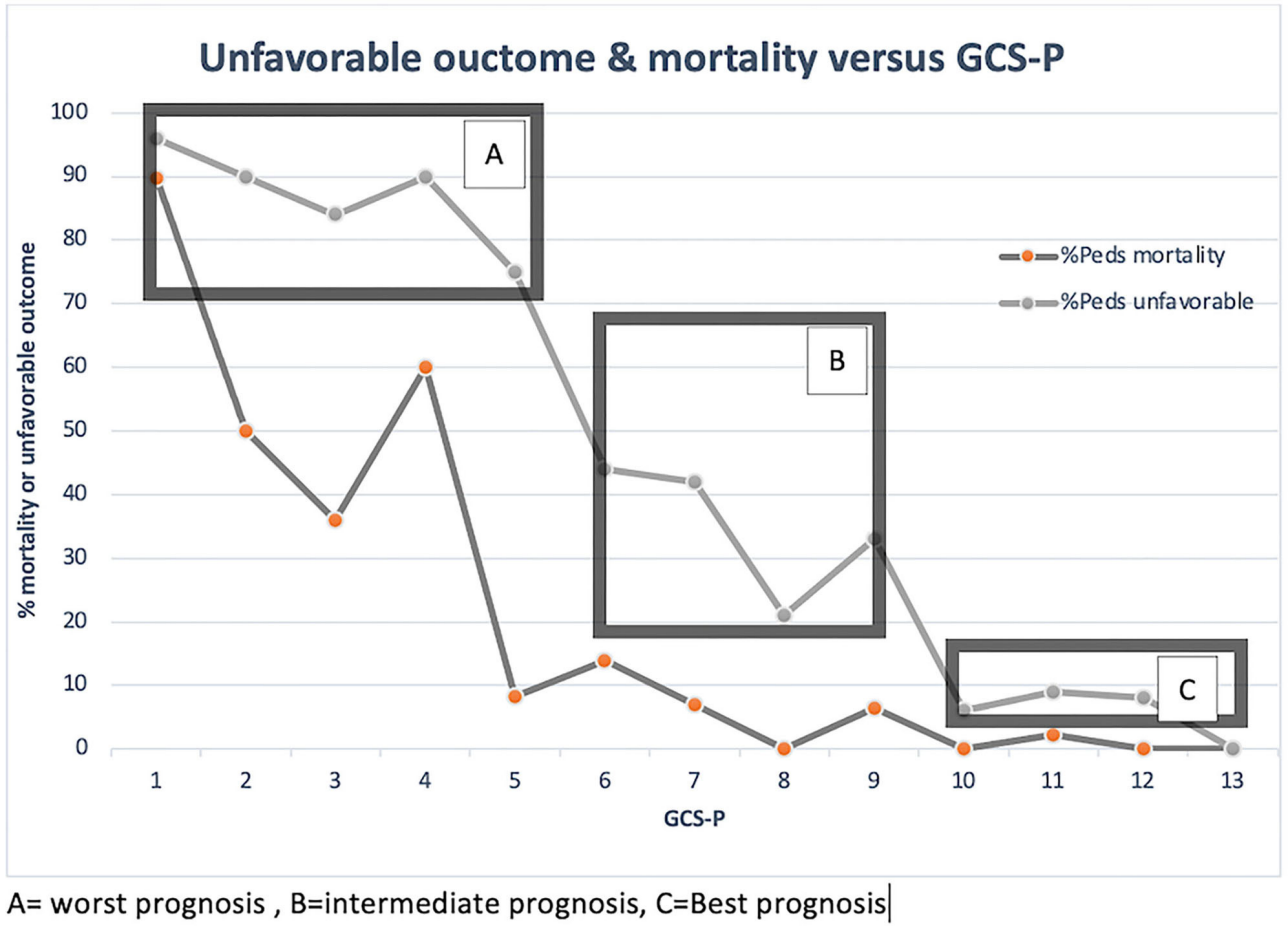
GOS Dichotomized and Mortality vs. GCS-P.

Higher GCS-P was associated with lower rates of mortality and unfavorable outcome (Table 1). In patients with a GCS-P of <5, the rate of unfavorable outcome was 75–95%, followed by a precipitous drop to 20–45% among patients with GCS-P 6–9, and a further significant reduction to <10% for patients with GCS-P 10–13 (Figure 2), indicating that there may be three prognosis groups after pediatric head injury with the utilization of GCS-P, best, intermediate, and worst prognosis.

#### CT image findings

The presence of subdural hematoma (36%, *p* < 0.001), intraparenchymal hemorrhage (46%, *p* < 0.05), and traumatic subarachnoid hemorrhage (36%, *p* < 0.05) on the CT head at presentation were associated with a modest increase in the unfavorable outcome. Intraventricular hemorrhage was associated with a more substantial increase in the rate of unfavorable outcomes (71%, *p* < 0.0001). Unfortunately, the low number of patients in this category (*N* = 20) limits our ability to investigate this finding in greater detail (Table 1).

### Performance of GCS, GCS-P, mechanism, age, and sex in predicting mortality and outcome

We compared the performance of GCS, GCS-P, motor score, and pupil reactivity in predicting mortality and unfavorable outcome in univariable (base) models (Table 3). Except for pupil reactivity alone, all metrics performed well in predicting mortality and unfavorable outcome, as defined by AUC > 0.85. However, GCS-P (mortality: AUC 0.995, CI 0.9342-0.9769; unfavorable outcome: AUC 0.8672, CI 0.8321-0.9024) did not significantly increase predictive power compared to using the GCS alone (mortality: AUC 0.9359, CI 0.9119-0.9599; unfavorable outcome: AUC 0.8596, CI 0.8241-0.8951).

**Table 3:** Predictive accuracy [Area Under the Curve (AUC) of the Receiver Operator Characteristic].

**6 month outcomes**				
	**Mortality**		**Unfavorable outcome**	
**Variables included in Model:**	**AUC (95% CI)**	**Somer's D-statistic**	**AUC (95% CI)**	**Somer's D-statistic**
GCS	0.9359 (0.9119–0.9599)	0.8717	0.8596 (0.8241–0.8951)	0.7192
GCSP	0.9556 (0.9342–0.9769)	0.9111	0.8672 (0.8321–0.9024)	0.7344
GCS Motor	0.9172 (0.8803–0.9543)	0.8345	0.8408 (08044–0.8771)	0.6816
PRS Score	0.8731 (0.8187–0.9275)	0.7462	0.7144 (0.6732–0.7557)	0.4289
GCS+age	0.9420 (0.9185–0.9655)	0.8840	0.8653 (0.8303–0.9003)	0.7306
GCSP+age	0.9576 (0.9372–0.9779)	0.9111	0.8726 (0.8380–0.9071)	0.7451
GCS Motor+age	0.9269 (0.8925–0.9613)	0.8539	0.8410 (0.8022–0.8798)	0.682
PRS Score+age	0.8656 (0.7963–0.9350)	0.7313	0.7201 (0.6639–0.7764)	0.4402
GCS+age+mechanism	0.9502 (0.9278–0.9725)	0.9003	0.8718 (0.8371–0.9065)	0.7436
GCSP+age+mechanism	0.9606 (0.9410–0.9800)	0.9210	0.8763 (0.8418–0.9108)	0.7526
GCS Motor+age+ mechanism	0.9341 (0.9016–0.9666)	0.8682	0.8558 (0.8193–0.8924)	0.7117
PRS Score+age+ mechanism	0.9009 (0.8529–0.9489)	0.8018	0.7839 (0.7376–0.8302)	0.5678
GCS+age+ mechanism+sex	0.9508 (0.9288–0.9728)	0.9016	0.8717 (0.8369–0.9064)	0.7433
GCSP+age+ mechanism+sex	0.9611 (0.9423–0.9799)	0.9221	0.8764 (0.8420–0.9109)	0.7529
GCS Motor+age+ mechanism+sex	0.9368 (0.9045–0.9691)	0.8736	0.8552 (0.8185–0.8920)	0.7105
PRS Score+age+ mechanism+sex	0.9094 (0.8646–0.9543)	0.8189	0.7858 (0.7397–0.8319)	0.5716

* *Somer's D measures the strength and direction of the association (closest to 1 best)*.

Multivariate models were then constructed using additional variables (age, mechanism of injury, and gender) in a stepwise fashion to assess the incremental prognostic value of each. Additions increased the predictive accuracy of the models only slightly, with the best performing model (GCS-P+age+mechanism+sex) accounting for 96.1% of the variability in mortality and 87.6% in unfavorable outcomes vs. 95.6% and 86.7, respectively.

We further explored sex- and age-related differences in mortality and unfavorable outcome after stratifying patients into moderate and severe TBI groups (Table 4). There were no statistically significant differences in outcomes by sex or age across injury severity strata, although there was a trend toward higher rates of mortality and unfavorable outcome among neonate males (*p* = 0.0940, 0.3043, respectively). We did not examine the distribution of injury mechanisms by age group due to an insufficient number of observations to generate meaningful comparisons.

**Table 4:** Outcomes by the severity of injury for TBI cohort (age and sex interaction).

	**Moderate head injury**			**Severe head injury**		
	***n***	**Mortality**	**Unfavorable**	***n***	**Mortality**	**Unfavorable**
**Male**
Neonates and infants	13	0 (0)	1 (7.7)	12	7 (58.3)	10 (83.3)
Toddler	24	0 (0)	2 (8.3)	18	6 (33.3)	10 (55.6)
Early childhood	45	1 (2.2)	4 (8.9)	19	5 (26.3)	9 (47.4)
Middle childhood	43	0 (0)	5 (11.6)	34	9 (26.5)	19 (55.9)
Early adolescence	65	0 (0)	5 (7.7)	69	14 (20.3)	35 (50.7)
*p*-value		0.6579	0.9683		0.0940	0.3043
**Female**
Neonates and infants	11	0 (0)	3 (27.3)	0	0 (0)	0 (0)
Toddler	12	0 (0)	2 (16.7)	14	3 (21.4)	7 (50.0)
Early childhood	22	0 (0)	0 (0)	11	1 (9.1)	7 (63.6)
Middle childhood	31	0 (0)	2 (6.5)	24	5 (20.8)	12 (50.0)
Early adolescence	32	0 (0)	3 (9.4)	21	8 (38.1)	14 (66.7)
*p*-value		0.9683	0.0847		0.3372	0.6268

*Severe Head Injury: GCS 3-8, Moderate Head Injury: GCS 9-13*.

*P-values correspond to the association of outcomes by age for males and females separately for each severity group*.

### Abusive head trauma subgroup analysis

The majority of patients with AHT (84%) were children <2 years of age. Although patients with AHT (*n* = 50) represented only 8.8% of the entire study cohort (*n* = 570), they comprised 23% of those who died. The mortality (36 vs. 11.3%, *p* < 0.0001, Tables 5, 6) and unfavorable outcome (56 vs. 29%, *p* < 0.0001, Table 6) were significantly higher in patients with AHT vs. the rest of the cohort. When compared to the AHT group, non-AHT patients had a better overall recovery: 66 vs. 44% were in the upper prognosis group and 22 vs. 52% were in the lower prognosis group.

**Table 5:** Patient outcome by GOS-E in TBI (non-AHT) vs. AHT.

**GOS-E outcome**	**TBI** ** (*n =* 520)**	**AHT** ** (*n =* 50)**
Upper good recovery	213 (40.9)	18 (36)
Lower good recovery	65 (12.5)	3 (6)
Upper moderate disability	63 (12.1)	1(2)
Lower moderate disability	29 (5.6)	0 (0)
Upper severe disability	33 (6.3)	2 (4)
Lower severe disability	53 (10.1)	8 (16)
Vegetative	5 (0.9)	0 (0)
Death	59 (11.3)	18 (36)

**Table 6:** AHT vs. TBI outcomes.

	**Total %**		**Mortality %**		**Unfavorable outcome %**	
**Severity**	**AHT**	**Non-AHT**	**AHT**	**Non-AHT**	**AHT**	**Non-AHT**
Moderate	40.0	47.9	20.0	0.4	40.0	10.4
Severe	58.0	42.7	48.3	26.1	69.0^*^	55.4^*^

* *not significant at.05 level*.

*Severe Head Injury: GCS 3-8, Moderate Head Injury: GCS 9-13*.

Furthermore, except for neonates (mean GCS 9), patients presenting with AHT had more severe injuries (mean GCS 7 vs. 9) and were more likely to have abnormal pupil reactivity (32 vs. 16%). After correcting for the severity of injury, the differences in outcomes of AHT patients persisted, suggesting a distinct relationship between GCS and outcome among the AHT cohort. The mortality rate of patients with severe AHT was almost double that of severe non-AHT (48 vs. 26%, *p* < 0.05). Among patients with moderate AHT, the mortality rate approached that of their severe non-AHT counterparts (20 vs. 26%, *p* < 0.05). The proportion of patients with unfavorable outcomes was higher among the AHT group regardless of severity, although only the moderate TBI group showed statistical significance. In patients with AHT and non-reactive pupils, the rates of mortality and unfavorable outcomes exceeded those of clinically similar non-AHT cohorts (87 and 100% vs. 73 and 89%, respectively: not statistically significant).

## Discussion

In one of the largest single institutional case series of pediatric patients with moderate and severe TBI, we examined the prognostic value of injury severity, age, and sex on mortality and unfavorable outcome at 6 months. Additionally, we explored the advantage of using a novel injury severity score (GCS-P) to predict mortality and 6-month outcomes. Our study demonstrated that GCS was the most powerful predictor of 6-month outcome, outperforming GCS-P, pupil score, and a number of well-established clinical and CT factors. This is important for clinicians seeking a reference by which to guide family counsel regarding short-term prognosis following injury. After stratifying patients by severity of injury based on GCS, no age- or sex-related effects were observed in our patient population.

### Review of age and pediatric TBI literature: Gaps in knowledge and our contribution

Prior studies have concluded that brain injury at an early age is not compensated for by the increased plasticity of the young brain, but rather is associated with a worse outcome because of increased vulnerability of the developing brain (28–30). For example, Levin et al. evaluated 155 children with severe TBI in three age groups (< 5, 5–11, and 11–18 years) and reported a mortality rate as high as 62% in the youngest age group 1 year after injury, higher than our estimate of 58% (31). In more recently published work by Sarnaik et al. the average uncorrected mortality rate in patients < 5 years of age was 14%, much lower than our findings and the reported work of Levin et al. (6, 32). In another study of 315 children, the mortality rate of children younger than 2 years of age with severe TBI was 47% (5). Although our mortality rate estimates appear somewhat higher than those reported in academic literature, it is important to note that all patients meeting the age/GCS/mechanism criteria, including those not considered for life-saving interventions and those who expired following resuscitation in the ED, were included in the study. Additionally, we tend to be aggressive with neurosurgical intervention in this patient population. Patients with a GCS of 3, bilateral non-reactive pupils, present brain stem reflexes, and surgical mass lesions or diffuse swelling are often taken for immediate neurosurgical intervention, while recognizing that the mortality or unfavorable outcome rates could be very high (32).

In our study, we also observed a trend toward worse outcomes in the neonatal group, although it was not statistically significant after controlling for GCS. However, the small sample size (only 6.9% of the study population) may explain the lack of statistical significance. Alternatively, earlier studies may have included neonates with AHT in their analysis, thus capturing the high mortality and unfavorable outcome rates associated with this mechanism of injury, rather than a true age-related or developmental effect. Our findings strongly indicate that patients with AHT need to remain a separate category in studies and potential clinical trials of patients with TBI.

### Review of sex and TBI: Gaps in knowledge/our contribution

Although some earlier studies have reported worse outcomes in female subjects (15), we did not find differences in outcomes by sex after correcting for the severity of injury. We did not subsequently stratify the sample according to the severity of the mechanism as the numbers in each sample would be very small, but there may be significant differences between the type and speed of the injuries encountered in male or female populations. Another factor may be risk-taking behaviors that may be associated with interactions between sex and mechanism, with males sustaining higher energy transfer injuries or lacking the use of protective devices. However, we do not have such details in our dataset (33). Differences in outcomes between sexes may be more evident later in the trajectory following injury and therefore continued tracking of outcomes for months to years is critical (13). In addition, recovery patterns in male and female populations may be different and the effects thereof are not as evident early after injury. However, these effects may not be detectable in the first months and only emerge years after head injury. Our study suggests that, at least in the first 6 months post-injury, our Center did not detect significant differences in male and female children as it pertained to TBI severity and outcome.

### Utilization of the GOS-E as an outcome measure

The GOS-E is a well-accepted scale to assess outcomes after pediatric TBI and performs well compared to other standardized pediatric assessment scales. The GOS-E assessment surveys multiple areas of functioning, including school/work performance, independence in and outside of the home, and maintenance of social relationships and interactions within the family. However, each of these data is collapsed into a single ordinal number that only very broadly describes the outcome of patients and does not assess the variability in performance that may exist between the different outcome domains and their specific impact on the patient, family resources, and relationships. While many assessment metrics have been reported in other outcome assessment studies, (34) we propose that a novel assessment of long-term functional outcome metrics is needed that can provide a meaningful assessment of the practical aspects of care that are required to support the recovering patient. Understanding these nuances of anticipated recovery is important for families as it may affect how they direct their resources. In our current study, the different outcome domains were not collected separately, but in an ongoing study, we are collecting long-term outcome data from this patient group and will record specific performance in the separate domains.

### Utilization of GCS/GCS-P scores in the pediatric population for outcome prediction

The GCS-P, a combination of the Glasgow Coma Scale score and the pupillary exam, was recently validated in a large adult study of TBI and performed better than the stand-alone GCS in predicting 6-month outcome after TBI (24). The GCS-P incorporates the two predictors that are consistently correlated with outcome across many studies, the GCS and pupil reactivity score, and collapses it into a single score that is intuitive to understand and use in the clinical setting. However, when testing our hypothesis and comparing the performance of the GCS-P to the GCS in our population, we did not find significant additional predictive power for the 6-month mortality of the GCS-P as compared to the GCS. In this same analysis, we also noted that the predictive power of the pupil score on its own was less than either the GCS or GCS-P, which may explain the absence of additional predictive power. To determine if GCS-P is a valid predictive tool in pediatric head injury, a formal validation study utilizing pediatric IMPACT data and including a full range of head-injured patients (mild-severe) will need to be done, an effort that is currently underway using our institutional data. In a larger analysis, we may also be able to explore the utility of stratifying three levels of initial injury severity in predicting a 6-month outcome with GCS-P as presented in our results. Overall, the stand-alone GCS had a very strong performance in predicting both mortality and unfavorable outcome (93 and 85%, respectively), and this should give clinicians confidence in presenting parents with the anticipated outcome 6 months from the injury.

Others have also assessed the predictive power of GCS for outcomes in pediatric patients. Abeytunge et al. studied 196 patients to develop a tool to predict the mortality of patients with severe TBI admitted to the Pediatric Intensive Care Unit (PICU) (35). They found that a pre-sedation GCS of 5 or less, a Rotterdam score of 3 or more, and a PTT value of more than 34.5 s were predictors of mortality with a combined positive predictive value of 94%. We did not include laboratory values but used each of the variables that make up the Rotterdam score in our study and entered these in the multi-variate analysis. The GCS alone had a similar positive predictive value in our study; however, the addition of other variables, whether secondary injuries or CT scores, did not add value. Notable is that we used the post-resuscitation GCS in our study, largely because of concern for inaccurate initial assessment en route to the hospital or in the trauma bay. The post-resuscitation GCS was captured from the neurosurgery consult notes and therefore obtained by neurosurgical personnel skilled in GCS scoring. In addition, it is common practice at our institution to hold sedation until an accurate GCS can be obtained and this number is typically reported in the consult notes. It is possible that capturing the GCS in this fashion may have reduced error and resulted in a stronger correlation with outcome.

### AHT—gaps in knowledge/our contribution

AHT in the pediatric population has been studied extensively, with particular emphasis on detection, prevention, and interventions (34, 36–38). Based on the review of current academic literature, AHT is associated with worse outcomes than other mechanisms of injury, although pathologic mechanisms responsible for these results are not fully elucidated (39). Some studies suggest that AHT is more likely to be associated with clinical factors predictive of poor outcomes such as intracerebral hemorrhage, injury at the craniocervical junction, cerebral edema, and ischemia (39). Alternatively, Miller Ferguson and colleagues suggested that some reported differences in mortality rates may be overstated as studies do not stratify patients appropriately by severity or treatment modality such as ICP monitoring, and children treated in centers committed to the following published guidelines with respect to ICP treatment/monitoring may represent a more appropriate study population (39). The strength of our study is that it is based on admissions to Level 1 trauma center, with consistent access to appropriate resources and modern guideline-based management of severe TBIs including ICP monitoring. Thus, our findings are more likely to reflect true clinical disparities in the study population rather than inconsistent clinical practices.

The literature suggests that boys are more likely to be victims of AHT, (34) and our study is consistent with these findings. We did not detect a difference in mortality and long-term outcomes by sex among this patient population. This is also consistent with the findings reported in other recent studies (36). Similar to prior studies, (34, 38) patients with AHT were significantly younger at presentation, with a median age of 10 months. Older age at the time of presentation was associated with higher mortality and worse overall recovery at 6 months.

While we have examined outcomes up to 6 months following injury, AHT tends to occur at a younger age and its full impact on development and function may not become apparent for many years (40). Deficits impacting daily functioning, learning, and behavior have been shown to emerge years later even among the patients who appear to have recovered in infancy (40). Eismann et al. found that among infants with AHT, overall cognitive development, fine motor function, and expressive language have all declined with age, with deficits detected in 23% of patients shortly after injury and 32% of patients 2 years later (40). By the age of 5 years, 47% of patients with AHT in infancy had developmental delays (41). Additional research with longer follow-up is needed to identify disparities in outcomes and prognostic indicators for AHT and non-AHT patients.

### Limitations of our study, applicability to other populations, and future directions

As alluded to above, a limitation of our study is the timing of outcome assessment. Although 6 months after injury is generally accepted as a long-term outcome measure and has been used as the primary time point for assessing outcome in many clinical trials for TBI, it has become increasingly clear that recovery from TBI may continue well after 6 months. In adult severe TBI, it has been reported that 43% of survivors improve from an unfavorable to a favorable outcome from 3 to 6 months, 36% from 6 to 12 months, 38% from 12 to 24 months, and 54% from 6 to 24 months (42). The recovery process in children is less well-understood and we are prospectively collecting the long-term outcome in this cohort of children to understand these changes and the timepoints after injury when they occur. Important additional questions to address in this population will include potential treatment strategies as well as access to rehabilitation or the return to school.

Our study is a single institution case series. Therefore, our findings may not be generalizable to the population of children with moderate and severe TBI. Children were managed at a well-established Level 1 trauma center using best practices and with a diverse catchment area of over 6 million people. Race has been noted as a potential modifier with sex, (15) but we did not include this in our analysis. All children were included in the study regardless of race or ethnicity and therefore are reflective of regional diversity. We included only children with a positive finding on a head CT in our analysis, therefore excluding a few children with moderate TBI and normal intracranial imaging. In this group, however, we aimed to understand the outcomes of all our patients. Future studies, particularly long-term assessments, should consider factors such as socioeconomic, race/ethnicity baseline GCS, and 6-month GOS-E.

We did not collect data on the pre-injury status of the child, such as school performance, test results, social functioning, and behavior. While we would anticipate that pre-existing abnormal functioning will affect the GOS-E outcome assessment, we were unable to test this hypothesis. In addition, socioeconomic status, social support, and other social determinants, such as gender identity, may influence post-injury recovery. Furthermore, pre-existing behavioral abnormalities may have directed some children into engaging in activities associated with higher risk and therefore more severe injuries. These factors may lead to worse outcomes but are not captured in our analyses.

We did not collect detailed data regarding patients' ICU course, including the incidence of secondary injuries such as intracranial hypertension or patient-specific intervention utilized by providers to treat these issues. The specifics of one's ICU course, including the frequency of ICP spikes and the effectiveness of associated interventions, may influence their 6-month outcome. While this association is outside the scope of this study, we will strive to incorporate these data into future studies.

Although this is one of the larger clinical series and it is adequately powered to study the proposed questions, the total number of patients is still relatively small and therefore small variations could significantly skew the data and observations of the study. In addition, we had 17% lost to follow-up in this patient sample, which may introduce selection bias. While our outcome assessment team pursues multiple avenues for follow-up, this could result in inaccurate assessments of association. A larger patient sample may more adequately address our hypotheses, but this would require a much longer follow-up with the potential for change in patterns of care over time or a multi-institutional study which may add significant institutional variation in the management of the patients.

## Conclusion

In this large single-center study, we found that sex had no value in predicting 6-month outcomes after moderate or severe blunt pediatric TBI. While neonates had a higher mortality rate than older children and adolescents, we found that no age group had a statistically significant higher rate of mortality or poor outcome relative to other age groups.

We found that the GCS continues to outperform other clinical and imaging predictors and strongly correlates with 6-month outcomes in children and adolescents with moderate and severe head injury. Our findings support its use to guide clinical decision-making and prognostication in addition to emphasizing the need to stratify head injuries for severity when undertaking outcome studies. While the GCS-P performed similarly in our patient population, we did not observe added benefit from the PRS score in predicting patient outcomes. We also found that pre-hospital secondary injuries, while not adding to the predictive value of the GCS, clearly have a significant impact on outcomes. Better identification and further analysis of occurrence and dose of secondary injury may aid in improved prognosis and development of future interventions.

These data highlight the critical need for long-term outcome studies in our pediatric population to confirm the injury and/or recovery trajectories and to continue to evaluate such factors, whether it may be age, sex, race, socioeconomic status, or even treatment strategies are the best predictors of outcomes. Moreover, identifying the critical predictors of outcome will help our clinical team to best communicate with families regarding long-term expectations as well as to identify those patients who might most benefit from specific treatment paradigms.

## Data availability statement

The raw data supporting the conclusions of this article will be made available by the authors, without undue reservation.

## Ethics statement

The studies involving human participants were reviewed and approved by UC Davis Institutional Review Board. Written informed consent from the participants' legal guardian/next of kin was not required to participate in this study in accordance with the national legislation and the institutional requirements.

## Author contributions

MZ and LK were involved in developing the concept, design, aims, and IRB submission and writing leads. MN played a critical role in experimental design and analysis as well as in generating the necessary figures. MZ, LK, MN, KN, and GG were all involved in data interpretation, writing, and editing of the manuscript. All authors contributed to the article and approved the submitted version.

## Funding

This study was financially supported by the UC Davis Department of Neurological Surgery.

## Conflict of interest

The authors declare that the research was conducted in the absence of any commercial or financial relationships that could be construed as a potential conflict of interest.

## Publisher's note

All claims expressed in this article are solely those of the authors and do not necessarily represent those of their affiliated organizations, or those of the publisher, the editors and the reviewers. Any product that may be evaluated in this article, or claim that may be made by its manufacturer, is not guaranteed or endorsed by the publisher.
